# Synthetic Chiral Derivatives of Xanthones: Biological Activities and Enantioselectivity Studies

**DOI:** 10.3390/molecules24040791

**Published:** 2019-02-22

**Authors:** Carla Fernandes, Maria Letícia Carraro, João Ribeiro, Joana Araújo, Maria Elizabeth Tiritan, Madalena M. M. Pinto

**Affiliations:** 1 Laboratório de Química Orgânica e Farmacêutica, Departamento de Ciências Químicas, Faculdade de Farmácia, Universidade do Porto, Rua de Jorge Viterbo Ferreira, 228, 4050-313 Porto, Portugal; leticiacarraro@hotmail.com (M.L.C.); joaobigi@gmail.com (J.R.); up201703236@ff.up.pt (J.A.); madalena@ff.up.pt (M.M.M.P.); 2 Interdisciplinary Centre of Marine and Environmental Research (CIIMAR), Edifício do Terminal de Cruzeiros do Porto de Leixões, Av. General Norton de Matos s/n, 4050-208 Matosinhos, Portugal; 3 Cooperativa de Ensino Superior, Politécnico e Universitário (CESPU), Instituto de Investigação e Formação Avançada em Ciências e Tecnologias da Saúde (IINFACTS), Rua Central de Gandra, 1317, 4585-116 Gandra PRD, Portugal

**Keywords:** synthetic xanthones, chiral derivatives of xanthones, bioactivities, enantioselectivity, enantiomeric purity

## Abstract

Many naturally occurring xanthones are chiral and present a wide range of biological and pharmacological activities. Some of them have been exhaustively studied and subsequently, obtained by synthesis. In order to obtain libraries of compounds for structure activity relationship (SAR) studies as well as to improve the biological activity, new bioactive analogues and derivatives inspired in natural prototypes were synthetized. Bioactive natural xanthones compromise a large structural multiplicity of compounds, including a diversity of chiral derivatives. Thus, recently an exponential interest in synthetic chiral derivatives of xanthones (CDXs) has been witnessed. The synthetic methodologies can afford structures that otherwise could not be reached within the natural products for biological activity and SAR studies. Another reason that justifies this trend is that both enantiomers can be obtained by using appropriate synthetic pathways, allowing the possibility to perform enantioselectivity studies. In this work, a literature review of synthetic CDXs is presented. The structures, the approaches used for their synthesis and the biological activities are described, emphasizing the enantioselectivity studies.

## 1. Introduction

In the last few years, the relationship between chirality and biological activity has been of increasing importance in Medicinal Chemistry [1]. Chirality can now be considered as one of the majors’ topics in the design, discovery, development and marketing of new drugs [2,3,4]. Enantiomers of drugs often present different behaviours within pharmacodynamics and/or pharmacokinetics events [5,6,7] as well as different levels of toxicity [8,9,10]. These differences makes in the majority of cases therapeutics with single enantiomers of unquestionable advantages [11]. 

The importance of enantioselective studies and the increase of chiral drugs in the pharmaceutical market upsurges each year due to the advantages in potency, efficacy, selectivity and safety associated with the use of single enantiomers. The advances in enantioselective synthesis [12,13,14,15] as well as enantioresolution methodologies [16,17,18,19] aligned to the stricter requirements from regulatory authorities to patent new chiral drug [20,21,22] boosted the research in this field. 

The development of methods to obtain and analyse both pure enantiomers has acquired crucial importance in the early stage of drug development, as biological and pharmacological activity evaluation of both enantiomers are required. Thus, despite the diversity of structures with different groups and positions at the base scaffolds, the library of compounds for structure-activity relationship (SAR) studies should also include stereoisomers for enantioselectivity evaluation. To perform that type of studies, it is necessary to obtain both enantiomers with very high enantiomeric purity. The improvement of chromatographic instrumentation and the development of efficient chiral stationary phases (CSPs) [23,24,25,26] have made liquid chromatography (LC) the first choice for determination of enantiomeric purity. 

Chemically, xanthones (9*H*-xanthen-9-ones) are compounds with an oxygen-containing dibenzo-γ-pyrone heterocyclic scaffold [27], being considered as a privileged structure [28,29]. Within this class of compounds a broad range of biological and pharmacological activities has been reported [30,31,32,33,34]. Additionally, other applications have been described for xanthone derivatives, such as preparation of fluorescent probes [35,36] or stationary phases for LC [23,24,37]. 

Many naturally occurring xanthones, isolated from terrestrial as well as marine sources, are chiral, and present interesting biological activities [38,39,40]. The biosynthetic pathway of xanthones only allows the presence of specific groups in particular positions of the xanthone scaffold, which is a limiting factor for structural diversity. For this reason, in order to enlarge chemical space in this field, total synthesis needs to be considered [41,42] allowing the access to structures that otherwise could not be reached only with natural product as a basis for molecular modification. Moreover, higher number of compounds can be obtained for SAR studies. 

## 2. Synthetic Chiral Derivatives of Xanthones

Recently, there has been an increase interest in new bioactive xanthones obtained by synthesis, particularly in chiral derivatives of xanthones (CDXs). Some reasons that can justify this trend were the importance of this class of compounds in Medicinal Chemistry align with the fact that nature usually gives only one enantiomer and the synthetic procedures allow the preparation of both enantiomers to explore the enantioselectivity in biological screening assays. The promising biological and pharmacological activities of some chiral members of this family, the clinical advantages of a single enantiomer than a racemate, the scarce examples of synthetic CDXs described, and the possibility to perform enantioselectivity studies strengthens the obtaining of new synthetic CDXs. This review aims to gather the research findings on synthetic CDXs, reporting their biological and pharmacological activities. The structures and the bioactivity differences associated to the stereochemistry of the CDXs (enantioselectivity) as well as their enantiomeric purity are highlighted.

### 2.1. Synthetic CDXs Inspired in Naturally Occurring Xanthones

Natural compounds have always been a source of inspiration for the discovery of new therapeutic agents [43]. Historically, the first proposed synthesis of a xanthone was achieved by Michael, in 1883, and later by Kostanecki and Nessler, in 1891, through the distillation of *O*-hydroxy-benzoic acid, acetic anhydride and a phenol [44,45], while the first total synthesis of a naturally occurring xanthone was a euxanthone, described by Ullmann and Panchaud, in 1906 [46]. Several synthetic CDXs analogues of natural xanthones are described. The biological and pharmacological activities evaluated are summarized in Table 1.

#### 2.1.1. Synthetic Xanthonolignoids

Xanthonolignoids are a natural class of compounds isolated from plants of the *Clusiaceae* family (*Guttiferae*) [47]. They possess a phenylpropane core linked to a xanthone scaffold by a dioxane ring, formed by radical oxidative coupling [48,49]. Natural xanthonolignoids include kielcorins, cadensins, subalatin, calophyllumins and gemixanthone [49]. The first xanthonolignoid described was based in 2,3,4-trioxygenated xanthone being isolated, in 1969, from *Kielmeyera species* [50].

Considering that xanthonolignoids are bioactive molecules and very interesting templates for molecular modifications, several xanthonolignoids were isolated and synthesized [49]. Initially the main goal of their synthesis was to help in the structure elucidation of this class of compounds but subsequently, also to improve their biological and physicochemical properties. Both classic synthesis and biomimetic approaches have been used to obtain xanthonolignoids, mainly kielcorin derivatives [49]. The total synthesis of kielcorin derivatives requires several steps and drastic reaction conditions while the biomimetic way is based on natural building blocks and is achieved by an oxidative coupling of a suitable dihydroxyxanthone and a cinnamyl alcohol derivative, in the presence of an oxidizing agent at room temperature [49].

**Figure 1:**
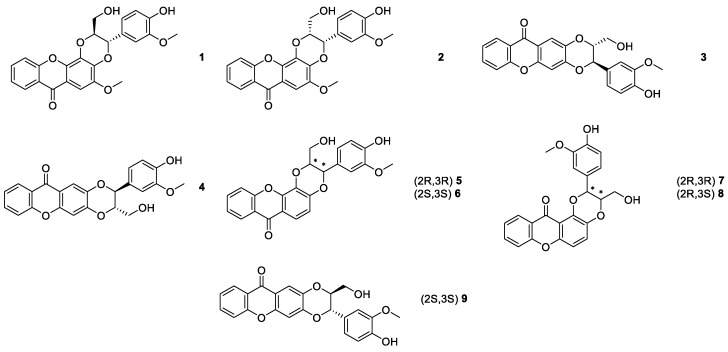
Structures of kielcorin (**1**) and synthetic derivatives **2**–**9**.

Pinto et al. [51], in 1987, reported the first biomimetic synthesis of xanthonolignoids of the kielcorin group, specifically kielcorin (**1**) and its stereoisomer, *cis*-kielcorin (**2**) (Figure 1). Both kielcorins **1** and **2** were also obtained by a classical method [52,53]. In 1999, by using a biomimetic approach, the synthesis of *trans*-kielcorin B (**3**) and *trans*-isokielcorin B (**4**) (Figure 1) was described [54]. 

To obtain related bioactive compounds with a kielcorin framework, other constitutional isomers were synthesized by our group, namely *trans*-kielcorin C (**5**), *trans*-kielcorin D (**6**), *trans*-isokielcorin D (**7**), *cis*-kielcorin C (**8**), and *trans*-kielcorin E (**9**) [55] (Figure 1). Once again, the synthetic approach, involving an oxidative coupling of coniferyl alcohol with an appropriate xanthone, was modelled on a biomimetic pathway. Different oxidizing agents were used (e.g., Ag_2_O, Ag_2_CO_3_, and K_3_[Fe(CN)_6_]) to investigate the oxidative coupling reactions. The synthesis of *trans*-kielcorin C (**5**) (also designated as demethoxykielcorin) using a classic approach was previously reported by Vishwakarma et al. [53].

Kielcorins **5**–**9** were evaluated for their *in vitro* effect on the growth of three human tumor cell lines, MCF-7 (breast), TK-10 (renal), UACC-62 (melanoma), and on the proliferation of human lymphocytes [56]. The growth inhibitory effect was moderate but dose-dependent and influenced by the isomerism of the tested compounds. The *trans*-kielcorins C (**5**) and D (**6**) were the most active. The inhibition of human lymphocyte proliferation induced by phytohemagglutinin (PHA) was detected [56]. The high potency and selectivity observed for these compounds suggested that kielcorins may be an important model for developing potent and isoform-selective protein kinase C (PKC) inhibitors [31]. Accordingly, kielcorins **5-9** revealed an effect compatible with PKC inhibition similar to that exhibited by the well-established PKC inhibitor chelerythrine [57]. The *trans*-kielcorins C (**5**) and E (**9**) were evaluated and demonstrated protectives effects against *tert*-butylhydroperoxide-induced toxicity in freshly isolated rat hepatocytes [58].

In order to study if the growth inhibitory effects of the kielcorins **5**–**9** depended on the stereochemistry, analytical LC methods using four carbamates of polysaccharide derivative CSPs and multimodal elution conditions were developed for their enantioresolution [59]. An amylose *tris*-3,5-dimethylphenylcarbamate CSP was chosen for a preparative resolution scale-up considering not only the highest enantioselectivity obtained for these chiral compounds, but also due to its low retention factors [59]. Consequently, the enantiomers of the kielcolins **5**–**7** and **9** were efficiently separated by chiral LC on a multimilligram scale. A solid-phase injection system was developed and combined with a closed loop recycling system to increase the productivity and recovery of the preparative process [60]. The enantiomeric purity was also measured being higher than 99% for each enantiomer, except for compound **5** [60].

The inhibitory activity of the racemates **5**–**7** and **9** as well as of the corresponding enantiomers on *in vitro* growth of the human breast adenocarcinoma cell line MCF-7 was evaluated and compared. The most evident enantioselectivity was noticed between the racemate of *trans*-kielcorin D (**6**) (inactive) and the active enantiomers (+)-**6** and (−)-**6** [60].

#### 2.1.2. Derivatives of Psorospermin

**Figure 2:**
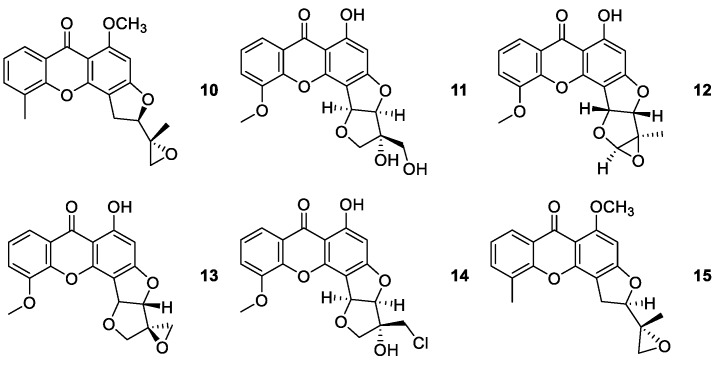
Structures of psorospermin (**10**) and synthetic derivatives **11**–**15**.

Psorospermin (**10**) was isolated from the bark and roots of the African plant *Psorospermum febrifugum*, in 1980 [61,62]. It is a natural fused tetracyclic xanthone containing two stereogenic centers with (2*R*,3’*R*)-stereochemistry and a reactive epoxide (Figure 2). The importance of the configuration and the functionality of the epoxydihydrofuran group for the *in vivo* activity have been evaluated [31,50]. The total synthesis of psorospermin (**10**) was reported for the first time in 2005, by obtaining the xanthone skeleton by the method of Grover et al. [62], including thirteen steps and with an overall yield of 1.7%. Psorospermin (**10**) revealed interesting biological activities showing antileukaemic, and antitumor activity in several human cell lines [31,62].

Additionally, the (*R*,*R*)-stereochemistry of psorospermin (**10**) gave optimum DNA alkylation and antitumor activity, although all four possible stereoisomers show topoisomerase II-dependent alkylation [63]. 

Two ring-constrained derivatives of psorospermin were also synthesized, namely, stereoisomer **12,** a ring-constrained (2*R*,3*R*)-form, and **13,** a ring-constrained (2*R*,3*S*)-compound (Figure 2) [63]. The chlorohydrin **14** retains psorospermin-like DNA alkylation characteristics despite its rigid structure and high affinity for DNA. The chlorohydrin **14** and epoxide **13** showed increased cytotoxicity against a range number of human tumor cell lines, compared to isohydroxypsorofebrin (**11**) [63]. 

Another study described the synthesis of two diastereisomeric pairs of *O*-5-methyl psorospermin and evaluation of *in vitro* activity against a range of solid and hematopoietic tumors. The diastereisomeric pair having the naturally occurring enantiomer (2*R*,3*R*) (**15**) (Figure 2) was the most active across all the cell lines tested. In subsequent studies using the four isomers of *O*-5-methyl psorospermin, the order of biological potency was (2*R*,3*R*) > (2*R*,3*S*) = (2*S*,3*R*) > (2*S*,3*S*) [64]. The compound (2*R*,3*R*) psorospermin (**15**) showed to be as effective as gemcitabine (chemotherapeutic drug) in slowing tumor growth *in vivo* in pancreatic cancer model [64]. 

#### 2.1.3. Derivatives of Muchimangins

**Figure 3:**
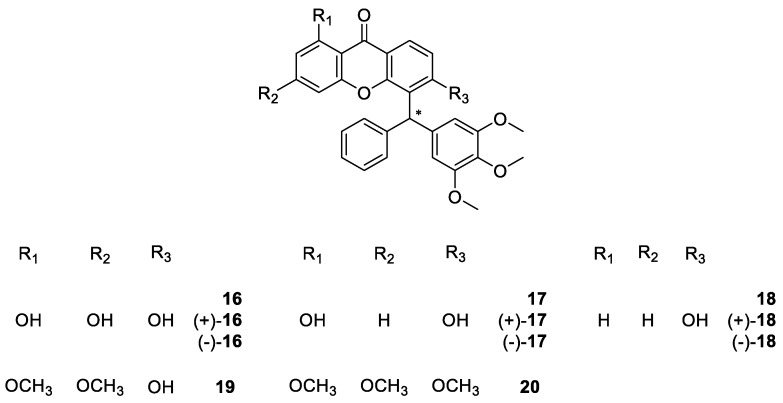
Structures of muchimangin derivatives **16**–**20**.

In many tribes and folk medicine use, plants and other organisms are commonly used to treat several conditions. For example, in Africa, the roots of *Securidaca longepedunculata* are used to treat sneezing, syphilis, gonorrhea, rheumatic pain, headache, feverish pain, malaria, sleeping sickness, among other conditions [65]. Muchimangins are a minor constituent of this specie and their biological activities have not been fully explored [66]. Dibwe et al. [67] reported the promising antiausteric activity of one natural occurring muchimangin against human pancreatic cancer PANC-1 cell line. Besides the anticancer promising activity, Kodama et al. [66] explored the antimicrobial activity of these structures and performed SAR studies. Accordingly, they synthesized several muchimangins derivatives **16**–**20** (Figure 3), and analyzed their antimicrobial activity. 

To synthesize the muchimangins derivatives **16**–**20**, they etherified commercially available 1,2,4-trihydroxybenzene with dimethyl sulfate, producing 1,2,4-trimethoxybenzene. Then, by acylation 2,4,5-trimethoxybenzophenone was obtained. This compound was further reduced to afford 2,4,5-trimethoxydiphenylmethanol, part of the muchimangin skeleton. Afterwards, the corresponding xanthone moiety was obtained using Eaton’s reagent. To finalize, both structural moieties were coupled by a Bronsted acid-catalyzed nucleophilic substitution, to produce the corresponding racemates [66]. In order to clarify the effect of chirality, Kodama et al. [66] separated the most promising derivatives using a CSP and to identify their optical rotation via polarimetry. 

The preliminary SAR studies suggested that the presence of a hydroxyl group at C-6 was important for the antibacterial activity. Moreover, enantioselectivity occurred for compound **18**, with the dextro (+) enantiomer being more active against *S. aureus* than the levo (-) enantiomer and the racemate [66]. 

#### 2.1.4. Derivatives of Mangiferin

**Figure 4:**
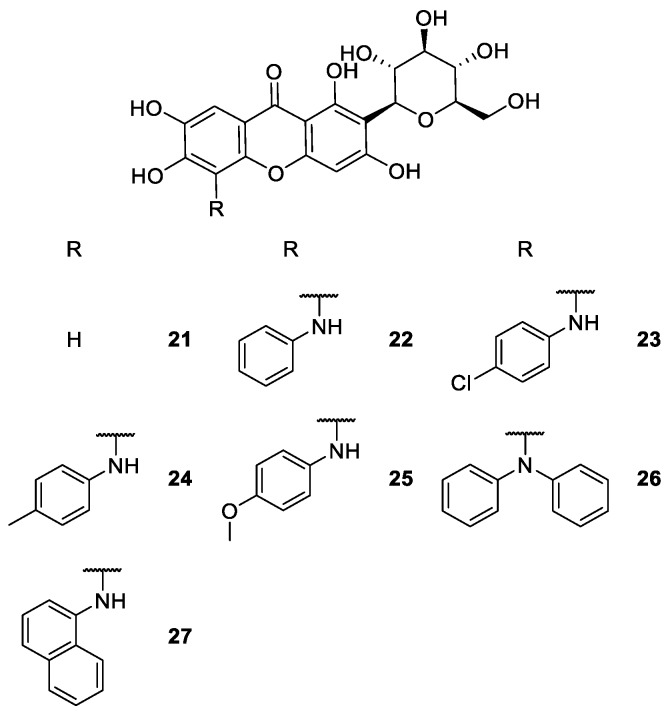
Structures of mangiferin (**21**) and some synthetic derivatives **22**–**27** with antipyretic and antimicrobial activities.

Mangiferin (**21**, Figure 4) is a natural occurring chiral xanthone with a large spectrum of biological activities, which have been explored for many years [68,69,70,71]. Several authors have compiled information about the biological properties of mangiferin and derivatives [72,73]. 

As previously reported by Araújo et al. [74], mangiferin derivatives present a large spectrum of antimicrobial activities. Singh et al. [75,76] developed new mangiferin derivatives **22**–**27** (Figure 4) and screened their antipyretic and antimicrobial activities. The synthetic strategy used equivalent molar proportions of mangiferin and an appropriate base (*R*-aromatic amine) at reflux to give the corresponding derivative. 

According to the results, it was suggested that the antipyretic activity may be attributed to the anti-inflammatory and antioxidant potential of mangiferin and its derivatives [76]. However, further investigations are required to understand the mechanism of action to prove its antipyretic activity. Regarding to the antimicrobial activity, the same compounds showed powerful inhibition of the growth of *S. virchow* and significant antibacterial activity against *B. pumilus* and *B. cereus*. On the other hand, all tested compounds revealed poor growth inhibition of *P. aeruginosa* and low antifungal activity [75].

**Figure 5:**
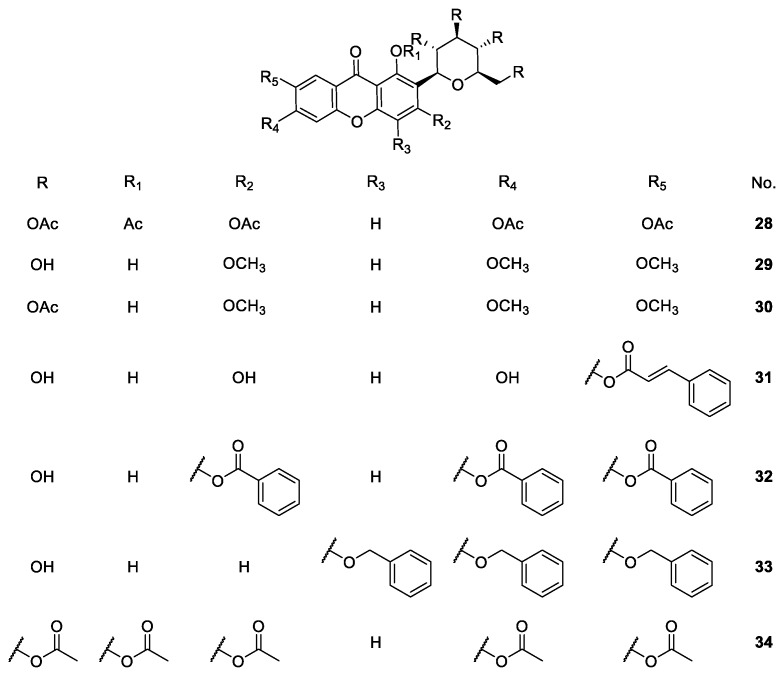
Structures of mangiferin derivatives **28**–**34** with antioxidant, anti-inflammatory and analgesic activities.

In other studies, the analgesic, antioxidant and anti-inflammatory activities of other mangiferin derivatives **28**–**34** (Figure 5) were explored [77,78]. Dar et al. [77] analyzed the analgesic and antioxidant activities of acetylated (**28**), methylated (**29**), and cinnamoylated (**30**) mangiferin, where compound **12** was acetylated to afford **14.** Mahendran et al. [78] observed the analgesic and anti-inflammatory activities of mangiferin with benzoyl (**31**), benzyl (**32**), and acetyl (**33**) groups.

The results demonstrated that mangiferin derivatives substituted with benzoyl (compound **32**) and acetyl (compound **34**) groups displayed better antioxidant activity than mangiferin (**21**) in lipid peroxidation, p-NDA, deoxyribose and alkaline DMSO assays, while neither compound had analgesic nor anti-inflammatory activities. In all of these methods, standard drugs showed better activity than mangiferin and its derivatives **28**–**34** [78]. 

**Figure 6:**
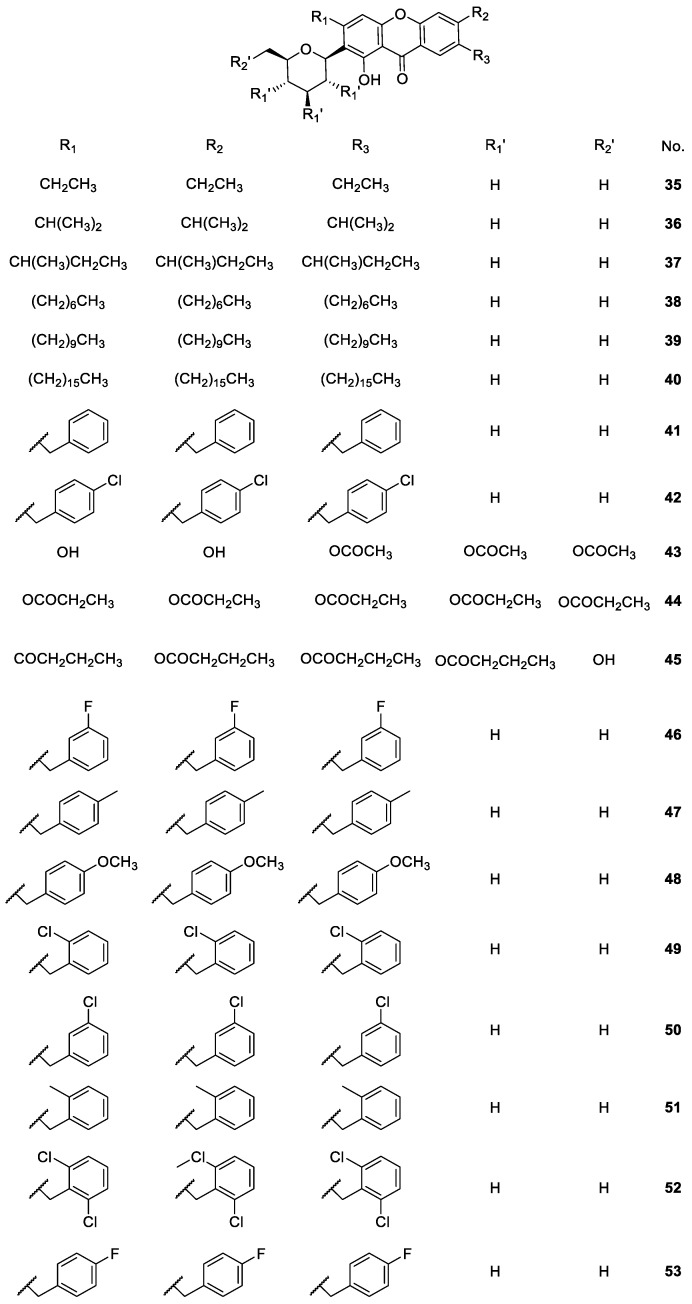
Structures of mangiferin derivatives **35**–**53** with antidiabetic activity.

Mangiferin (**21**) is also known to possess antidiabetic activity [79,80]. This biological activity was further investigated for other mangiferin derivatives **35**–**42** and **46**–**53**, by Hu et al. [81,82] and **43**–**45** by Li et al. [83] (Figure 6). These works evaluated the properties of mangiferin derivatives as protein tyrosine phosphatase 1B (PTP1B) inhibitors in order to demonstrate their hypoglycaemic activity. The PTP1B has an important role in type 2 diabetes and obesity [84], which is primarily responsible for the dephosphorylation of the activated insulin receptor and thus downregulates insulin signalling [85]. For this reason, PTP1B inhibitors are a good strategy for diabetes mellitus treatment. 

According to Hu et al. [81,82], mangiferin (**21**) is a weak PTP1B inhibitor, whereas some derivatives such as **36**, **38** and **39** showed good inhibition of this protein. The SAR studies suggested that the substitution of free hydroxyl at C-3, C-6, C-7 of mangiferin (**21**) remarkably enhanced the inhibition, and the mono- or dichloro benzylated derivatives displayed better inhibitory activity than other groups [81,82]. However, further modification and biological studies are still in progress [81].

Li et al. [83] also demonstrated that the esterified-derivatives of mangiferin **43**–**45** (Figure 6) could repair damaged islet cells, and had higher lipid-solubility, and more potent hypoglycaemic activity than the mangiferin (**21**). The SAR studies indicated that the larger the esterification moieties or the higher lipid-solubility, the more potent hypoglycaemic activity was displayed by the derivative. Thus, esterification proved to be an effective way to improve the activity of mangiferin as a potential antidiabetic drug [83].

**Figure 7:**
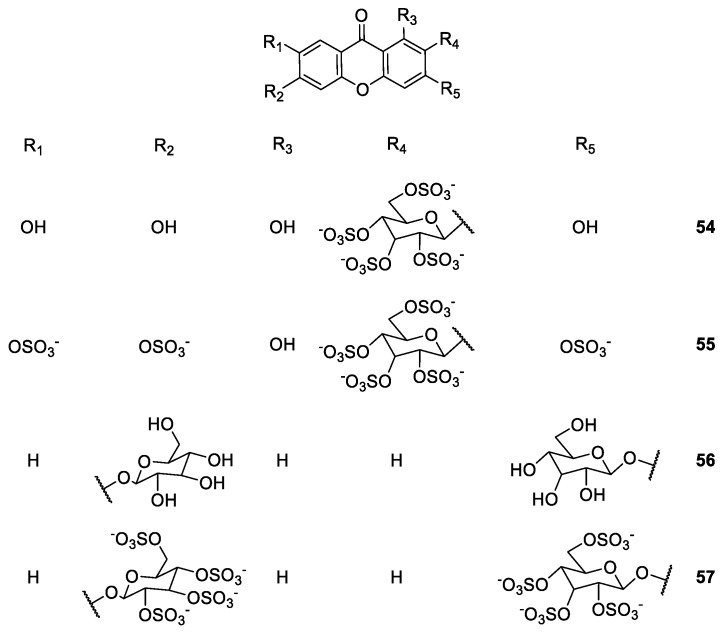
Structures of mangiferin derivatives **54**–**57** with anticoagulant and antiplatelet activities.

Correia-da-Silva et al. [86] developed new sulfated xanthones **54**–**57**, inspired by the mangiferin scaffold, to study their anticoagulant and antiplatelet properties (Figure 7). The synthetic approach included the sulfation of commercially available mangiferin affording mangiferin-2′,3′,4′,6′-tetrasulfate. It was found that an increase of the quantities of sulfating agent furnished the 2′,3,3′,4′,6,6′,7-heptasulfated derivative. The precursor of the other derivatives could be a suitable xanthone scaffold, where 3,6-(*O*-β-glucopyranosyl)xanthone was obtained after deprotection of the glycosylated xanthone 3,6-(2,3,4,6-tetra-*O*-acetyl-β-D-glucopyranosyl)xanthone. The sulfation of 3,6-dihydroxyxanthone allowed preparation of persulfated 3,6-(O-β-glucopyranosyl)xanthone. Two polysulfated xanthonosides proved to be inhibitors of thrombosis, combining anticoagulant and antiplatelet effects in a single molecule [86].

#### 2.1.5. Derivatives of α-Mangostin

One of the most studied xanthones found in Nature is α-mangostin, isolated from tropical fruits of *Garcinia mangostana* which have been used for centuries in folk medicine to treat many conditions [87,88]. Several studies have reported its anticancer and antimicrobial activities, among others, which have prompted researchers all over the world to synthesize diverse derivatives [74,87,88,89,90,91,92,93,94].

**Figure 8:**
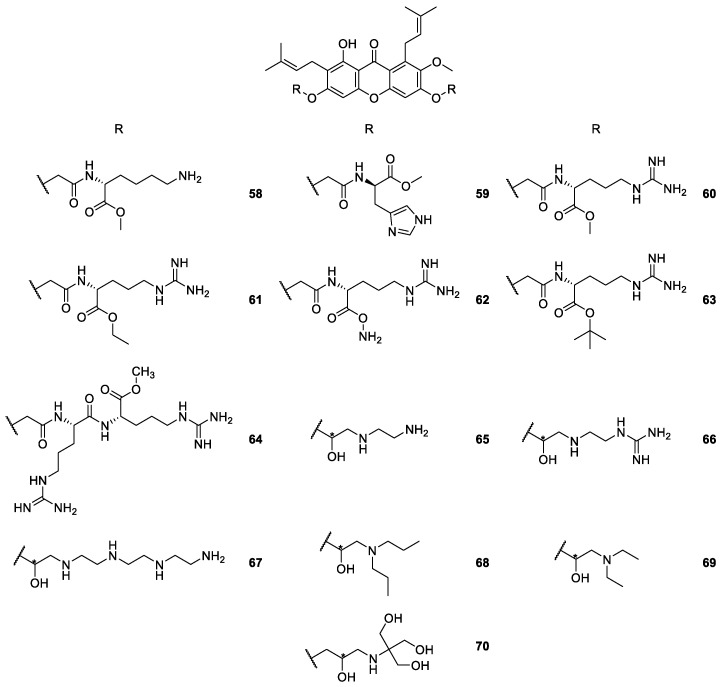
Structures of derivatives of α-mangostin **58**–**70**.

One strategy concerned cationic antimicrobial peptides (CAMPs),amphipathic structures whose hydrophobic moiety penetrates the membrane core, while the cationic residues disrupt bacterial membranes [95,96,97,98]. Due to the manufacturing costs and poor stability of peptides, Koh et al. [95,99] developed small-molecules **58**–**70** with CAMP characteristics by combining the α-mangostin core with basic amino acids moieties (Figure 8). The purpose of the work was to confirm if lipophilic chains enhance the membrane permeability and to examine the role of the cationic moieties conjugating the xanthone scaffold with basic amino acids [99]. This strategy was also used to develop new anti-mycobacterial derivatives **65**–**70** [95], and by Lin et al. [97] for studies seeking new antimicrobial and hemolytic compounds.

According to the results, the amphiphilic xanthone derivatives **65**–**70** (Figure 8) possessed promising mycobacterial activity without resistance mechanisms, which may contribute to the development of an entirely new class of therapeutics for tuberculosis [95]. Besides the interesting activity of these compounds, the cationic and hydrophobic moieties enhanced the water-solubility, and also lead to high membrane selectivity and excellent antibacterial activity against Gram-positive bacterial strains, including methicillin-resistant Staphylococcus aureaus and vancomycin-resistant Enterococcus. It is important to point out that the membrane selectivity of these compounds was higher than several membrane-active antimicrobial agents in clinical trials [97,99].

#### 2.1.6. Derivatives of Caged Xanthones

**Figure 9:**
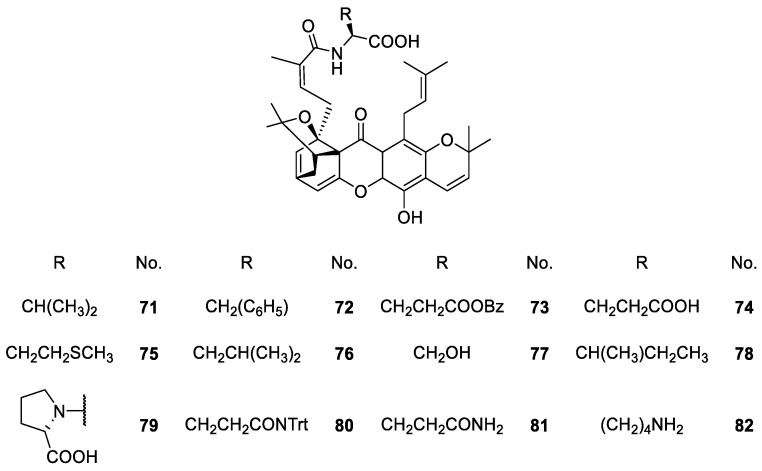
Structures of caged xanthones **71**–**82** with antimicrobial activity.

**Figure 10:**
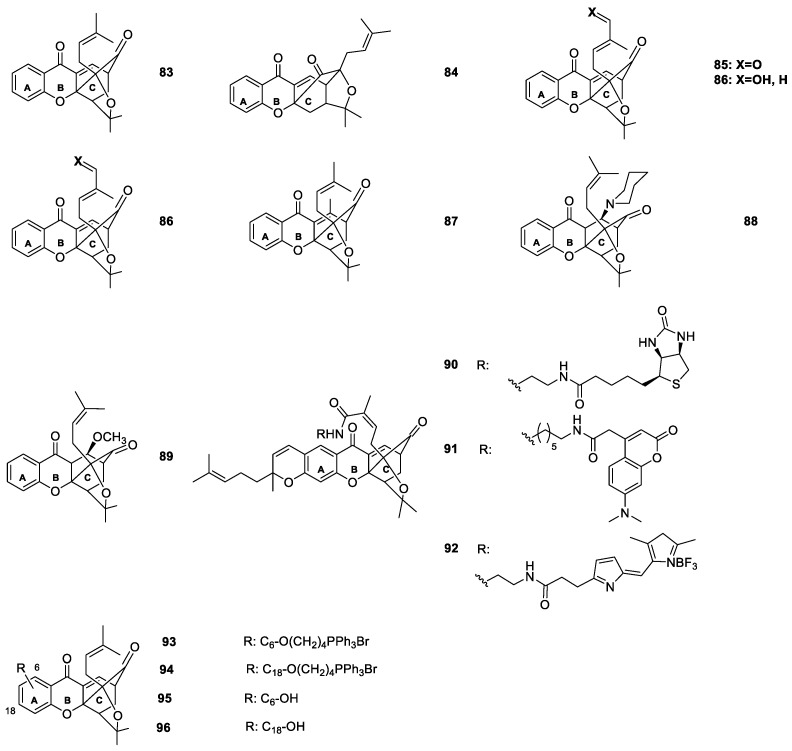
Structures of caged xanthone **83**–**96**, with antimalarial and antitumor activities.

**Figure 11:**
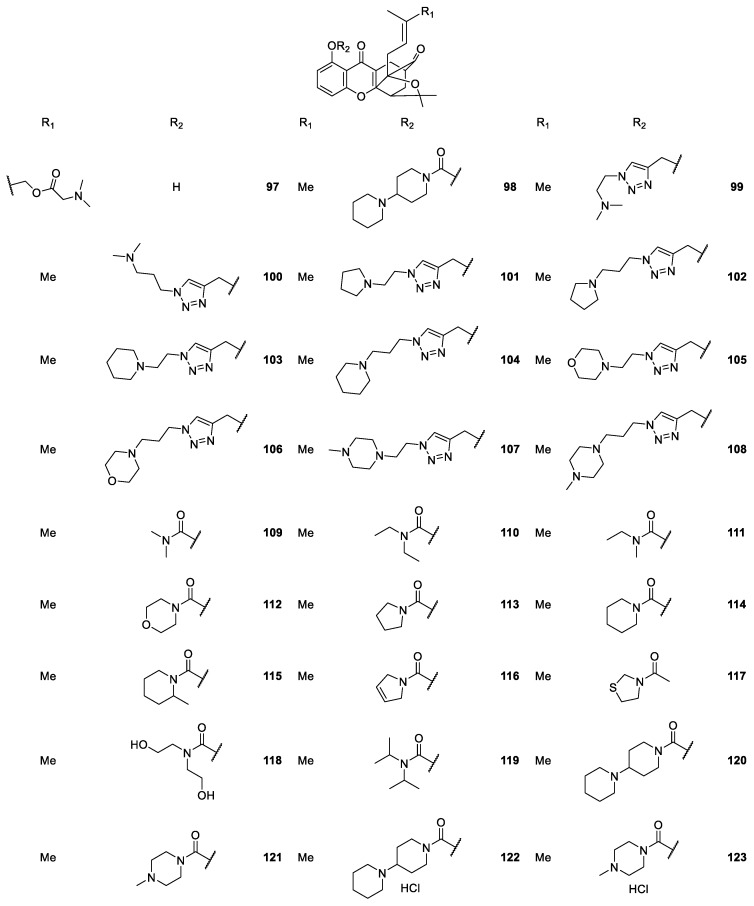
Structures of caged xanthones **97**–**123**, with antitumor activity.

**Figure 12:**
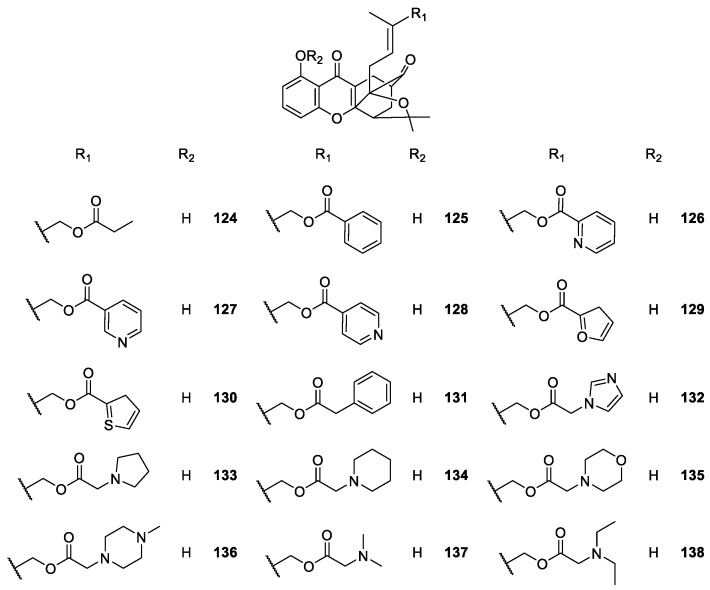
Structures of caged xanthones **124**–**138**, with antitumor activity.

**Figure 13:**
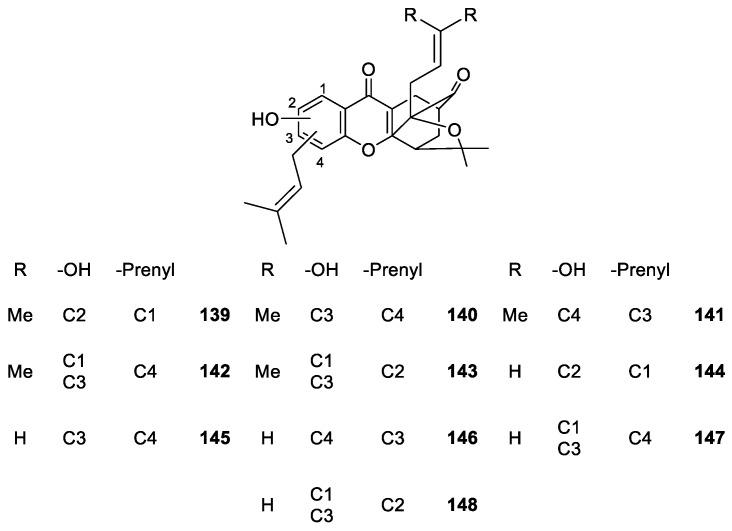
Structures of caged xanthones **139**–**148**, with antitumor activity.

**Figure 14:**
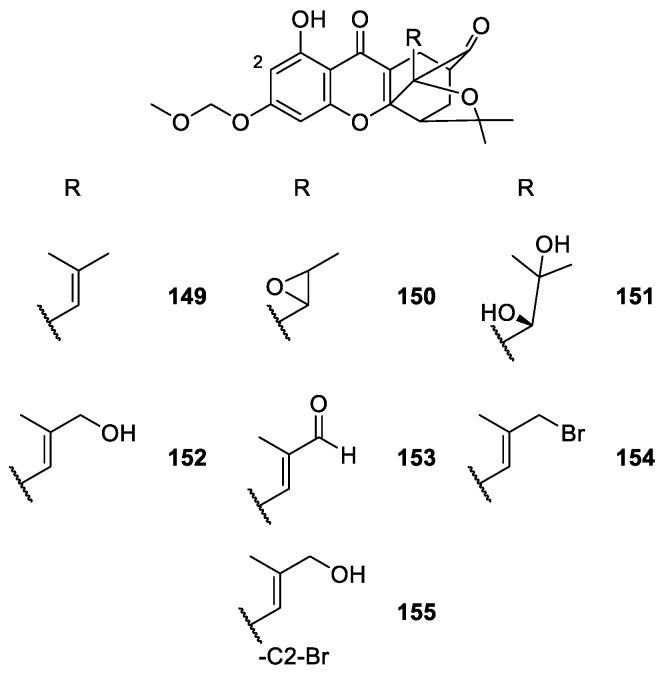
Structures of gambogic acid derivatives **149**–**155**, with antitumoral activity.

The *Garcinia* genus contains caged xanthones which mainly occur in a few species like *G. morella*, *G. hanburyi*, *G. bracteata*, *G. gaudichaudii*, and *G. scortechinii*, widely distributed in Southeast Asia [100,101,102,103]. The caged core is responsible for the vast range of bioactivities of this class of compounds, such as anti-viral, and antibacterial effects, among others [104,105,106,107]. Many reports have described the potential antitumor activity of gambogic and morrelic acids [100,101,104,105,107,108]. According to this, many caged xanthones **71**–**155** have been synthesized and studied through the last years, with a diversity of purposes (Figure 9, Figure 10, Figure 11, Figure 12, Figure 13 and Figure 14).

Chaiyakunvat et al. [105] inspired by the biological properties of caged xanthones, synthesized a few morrelic acid derivatives and evaluated their antimicrobial activity. They started with the synthesis of methylated morrellic acid and, afterwards, they synthesized derivatives **71**–**82** with amino acid moieties via solid-phase synthesis (Figure 9).

The morellic acid derivatives showing more inhibition of bacterial growth were the ones with an amino acid-containing hydrophobic side chain like **71**, **72**, **76**, **78** and **79** (Figure 9) [105]. This is in agreement with previous reports where the antimicrobial activity was higher in the structures with hydrophobic and/or aromatic amino acids [99,105]. 

Theodorakis et al. [106,109,110,111] synthesized new caged xanthones and studied their properties. They developed a Claisen/Diels–Alder reaction cascade that, in combination with a Pd(0)-catalyzed reverse prenylation, provided a rapid and efficient access to the caged xanthone pharmacophore. Afterwards, various A-ring oxygenated derivatives of cluvenone (**83**) were further synthesized and analyzed (Figure 10) [106,109,110,111].

The SAR studies showed that their activity could be substantially improved by attaching a triphenylphosphonium group at the A ring of the caged xanthone. Derivatives **93** and **94** (Figure 10) were found to be highly effective as antimalarials against *Plasmodium falciparum* [106]. The conjugation of these compounds with a phosphonium salt improved their efficacy, resulting in lead compounds with a promising therapeutic window [106]. It was suggested that, further modification of the caged xanthone could increase the selective cytotoxicity and lead to a promising lead candidate [106].

Cluvenone (**83**) was also reported to induce cell death via apoptosis, presenting similar cytotoxicity in multidrug-resistant and sensitive leukemia cells [109,110]. The caged xanthone derivatives proved to be active with cytotoxicity at low to sub-micromolar concentrations in solid and non-solid tumor cell lines, respectively. Additionally, they induced apoptosis in HUVE cells. Remarkably, similar IC_50_ values were obtained for the compounds tested in the HL-60 and HL-60/ADR cell lines, suggesting that these compounds were not subject to a drug resistance mechanism. Therefore, it was suggested that members of this family of compounds may have therapeutic potential in relapsed cancers typically resistant to standard chemotherapeutic agents. In addition, the cytotoxicity observed in HUVE cells suggested that these compounds may be interesting leads for the development of new angiogenesis inhibitors [111]. Elbel et al. [112] synthesized selected A-ring hydroxylated analogues and evaluated their effect on cell growth, mitochondrial fragmentation, mitochondriotoxicity and Hsp90 client protein degradation. They found out that both the C6 and C18 hydroxylated cluvenones inhibited the growth of CEM cells at low micromolar concentrations and induced cell death via the mitochondrial pathway. In addition, cluvenone (**83**) and the hydroxylated cluvenones induced Hsp90-dependent protein client degradation at low micromolar concentrations [112].

Zhang et al. [113,114,115,116] synthesized a series of caged xanthone derivatives to improve the physicochemical properties and *in vivo* cytotoxic potency. For that, they relied on MAD28 synthesis and characterization of the derivatives. The structural modifications revealed that the presence of a carbamate moiety was useful for obtaining comparable cytotoxicity and improved aqueous solubility and permeability (Figure 11).

It is important to highlight that compound **137** (named DDO-6306, Figure 12) displays growth inhibition in Heps transplanted mice, and is now undergoing further evaluation as a candidate for cancer chemotherapy [115]. In a more recent study, compound **105** (Figure 11), considered as the lead compound and called MAD28, successfully led to the discovery of a novel series of natural-product-like triazole-bearing caged xanthones with improved drug-like properties as orally-active antitumor agents *in vivo* [115].

Regarding the caged xanthone derivatives containing carbamate scaffolds **109**–**123** (Figure 11), the results showed a potent antiproliferative activity and good physicochemical properties, which contributed to improving their *in vivo* activities. The compound **122** (DDO-6337) showed moderate inhibitory activity toward Hsp90 ATPase and resulted in the degradation of Hsp90 client proteins, such as HIF-1, which ultimately contributed to its antitumor and anti-angiogenesis activities [116].

Compounds **140**–**143** (Figure 13) exhibited micromolar inhibition against several cancer cell lines. Some interesting SAR considerations have been highlighted, such as the importance of the periphery gem-dimethyl groups in maintaining the anti-tumor activity, the effect of the substituent at C-1 position of B-ring on activity, since hydroxyl group at C-1 position enhanced the potency while prenyl group reduces it, and, that the change of hydroxyl or prenyl groups in carbons C-2, C-3 and C-4 had no significant effect on the anti-tumor activity. These events indicated that referred sites can be used to improve drug-like properties [114].

In another study, Miao et al. [117] developed small molecule entities inspired by the structure of gambogic acid. They focused on modifications of the prenyl moiety of the caged xanthones which led to synthesize seven derivatives **149**–**155** (Figure 14), which were tested for anti-tumor activity [117].

The SAR studies suggested that compounds **151**, **153**, and **154** showed similar cytotoxicity to gambogic acid against A549 cells, whereas compounds **149**–**151** and **152** were less active than gambogic acid. Although these experiments were preliminary, the results suggested that promising agents with anti-tumor activities could be obtained by modification at C-2 position of the B ring and at C-21/22 or C-23 position of the prenyl group in the caged scaffold. The formation of dihydroxy and epoxy groups of the double bond at C-21/22 and the introduction of an electron-withdrawing group at C-23 evidently affected the anti-proliferation activity [117]. In Table 1 a summary of the synthetic CDXs inspired in natural xanthones and their described biological activities are presented, as well as the associated references. 

### 2.2. Synthetic CDXs Obtained by Binding/Coupling Chiral Moieties to the Xanthone Scaffold

Another approach to acquire synthetic CDXs is by binding chiral moieties to the xanthone scaffold using different strategies. The biological and pharmacological activities evaluated of the various CDXs obtained by this strategy are included in Table 2.

#### 2.2.1. XAA and DMXAA Analogues

**Figure 15:**
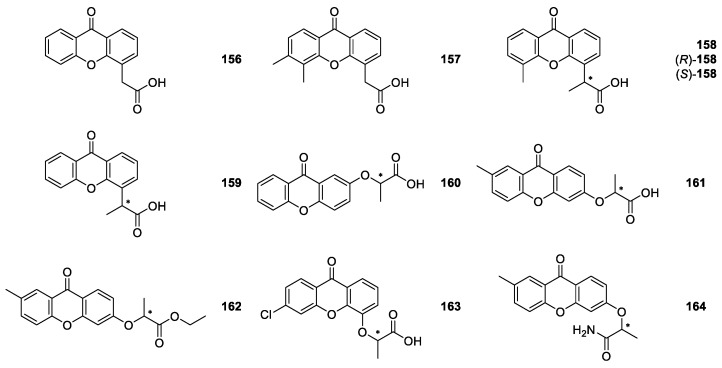
Structures of XAA (**156**), DMXAA (**157**) and chiral analogues **158**–**164**.

DMXAA (5,6-dimethylxanthone-4-acetic acid, vadimezan, ASA404, **157**, Figure 15) is a simple carboxylated xanthone discovered by SAR studies involving a series of xanthone-4-acetic acids (XAA, **156**, Figure 15) related to the parent compound flavone acetic acid [121]. DMXAA is one of the most studied xanthones considering not only its remarkable pharmacological profile [122,123,124,125,126,127,128,129,130,131], but also its physicochemical and pharmacokinetic properties [130,132,133]. It is a tumor vascular-disrupting agent leading to a fast, vascular collapse and tumor necrosis by immunomodulation and cytokines induction. DMXAA (**157**) also demonstrated antiviral [134], antiplatelet and antithrombotic [135] activities. This synthetic xanthone is not chiral but, it is evident that structurally and from a biological activity perspective, it may be an attractive scaffold for the development of other bioactive analogues and derivatives.

Rewcastle et al. [136], in 1991, synthetized the DMXAA chiral analogues 2-(5-methyl-9-oxo-9*H*-xanthen-4-yl)propanoic acid (**158**) and 2-(9-oxo-9*H*-xanthen-4-yl)propanoic acid (**159**) as racemates (Figure 15). CDX **159** was synthetized via bromination of 4-ethylxanthenone followed by conversion of the resulting 4-(1-bromoethyl)xanthenone to compound **159** via the nitrile. CDX **158** was obtained by reaction of 2′-hydroxyacetophenone with benzyl chloride under phase-transfer conditions, giving 2′-benzyloxyacetophenone, which was converted successively to an alcohol, using NaBH_4_, and a chloride, with anhydrous CaCl_2_ and HCl. After three reaction steps, 2-(2-hydroxyphenyl)propanoic acid was obtained and then reacted with 2-iodo-3-methylbenzoic acid via a copper/TDA-catalysed condensation. Finally, the resulting diacid was ring-closed using H_2_SO_4_ to give the racemic compound **158**. For this CDX both enantiomers were separated by indirect method employing (*R*)-(−)-pantolactone as chiral resolving agent. The obtained mixture of diastereomers was separated by chromatography on silica gel. Further, hydrolysis of the esters under non-enolizing conditions afforded both enantiomers, (*S*)-(+)-**158** and (*R*)-(−)-**158** (Figure 15) [136].

The racemic compounds **158** and **159**, as well as enantiomers (*S*)-(+)-**158** and (*R*)-(-)-**158** were tested in *in vitro* and *in vivo* tumor assays [136]. It was found that all the compounds were active. Moreover, enantioselectivity was observed, being the *S*-(+)-enantiomer much more dose-dependent than the *R*-(-)-enantiomer. It was suggested that the enantiomers have different intrinsic activities, rather than differing in their metabolism [136]. CDX **159** had been tested previously as anti-inflammatory agent [137].

Marona et al. reported the synthesis [138] of three new chiral analogues of XAA **160**–**162** (Figure 15) and the evaluation of their cytotoxicity against J7774A.1 cells [139]. Compounds **160** and **161** were obtained by condensation of 2-hydroxyxanthone and 2-methyl-6-hydroxyxanthone, respectively, with α-bromopropionic acid and compound **162** by esterification of compound **161** [138]. Regarding the biological activity tested, it was found that all CDXs showed weak cytotoxicity [139].

Recently, Zelaszczyk et al. [140] synthesized two new chiral XAA derivatives **163** and **164** (Figure 15) by the reaction of 3-hydroxyxanthone with ethyl 2-bromopropanoate, followed by ester hydrolysis [140]. These compounds were found to have anti-inflammatory and analgesic activities [34,140].

#### 2.2.2. Synthetic Aminoalkanolic CDXs

**Figure 16:**
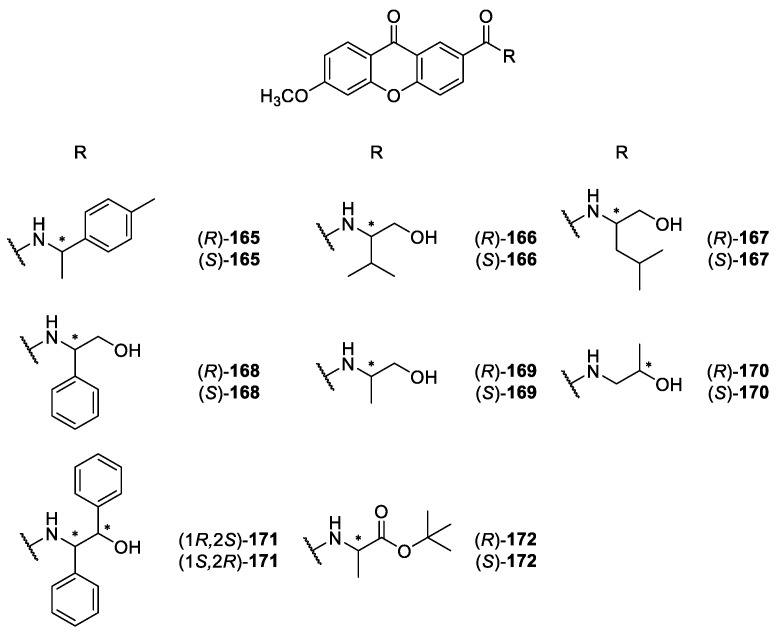
Structures of aminoalkanolic CDXs **166**–**171** and analogues **165** and **172**.

**Figure 17:**
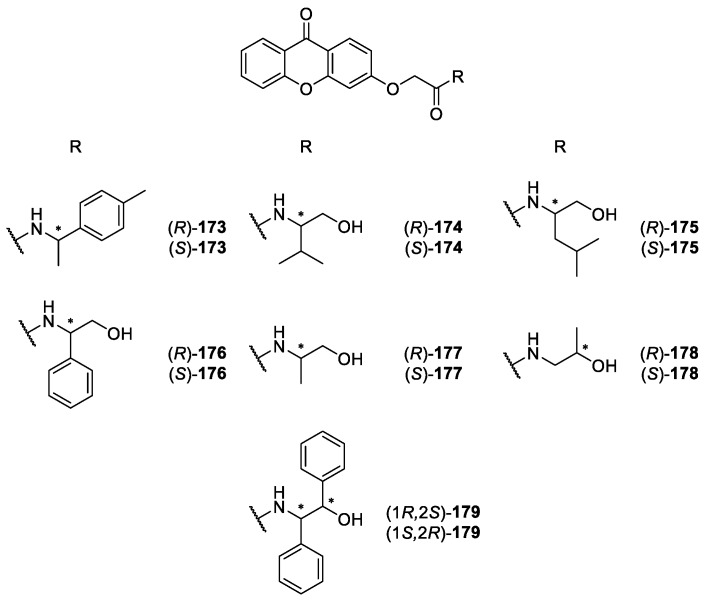
Structures of aminoalkanolic CDXs **174**–**179** and analogue **173**.

Our group has a vast experience in synthesis and biological/pharmacological activity evaluation of xanthone derivatives [86,141,142,143,144,145,146,147] and, recently, reported the synthesis of a library of CDXs **165-179** in an enantiomerically pure form (Figure 16 and Figure 17) [148,149]. Among the synthesized CDXs, the compounds **166-171** and **174**–**179** are aminoalkanolic, while CDXs **165, 172** and **173** comprise of simple amines with a *p*-tolyl moiety (compounds **165** and **173**) or an aminoester (compound **172**) [148,149].

Considering that carboxyxanthone derivatives are suitable molecular entities to perform molecular modifications to obtain new bioactive derivatives [34], the synthesis of all CDXs **165-179** were achieved by using two carboxyxanthone derivatives as substrates, namely 6-methoxy-9-oxo-9*H*-xanthene-2-carboxylic acid and 2-((9-oxo-9*H*-xanthen-3-yl)oxy)acetic acid. The synthetic strategy used was the coupling of the carboxyxanthone derivatives with both enantiomers of eight commercially available chiral building blocks, using *O*-(benzotriazol-1-yl)-*N*,*N*,*N*′,*N*′-tetramethyluronium tetrafluoroborate (TBTU) as coupling reagent [148,149]. TBTU has been widely used as efficient reagent for the synthesis of diverse classes of compounds, including peptides [150], esters [151,152], phenylhydrazides [153], acid azides [154], among others. However, this was the first report of the use of TBTU to synthesize CDXs [149]. All used commercial chiral blocks included both enantiomers of enantiomerically pure building blocks with no tendency towards racemization or enantiomeric interconversion and having a primary amine as reactive group for amide formation. Amino alcohols, amines, and amino esters were selected. The coupling reactions were performed at room temperature, showing short reactions times and excellent yields (ranging from 94% to 99%) [148,149]. The synthetic methodology used to obtain the referred CDXs provided to be very efficient, broad-scope applicability, and operationally simplest. Moreover, it was found that the synthesis of the CDXs was easily scaled-up for both enantiomers in order to obtain available quantities for biological and pharmacological assays as well as other applications.

LC using different types of CSPs, namely polysaccharide-based [149], macrocyclic antibiotics [155,156], and Pirkle-type [157,158] was used for enantioresolution studies and determination of the enantiomeric purity of the synthesized CDXs. The enantioselective LC method using polysaccharide-based CSPs under multimodal elution conditions afforded very high resolutions with short chromatographic runs. The best performances were achieved on amylose *tris*-3,5-dimethylphenylcarbamate stationary phase under polar organic elution conditions. The resolution achieved allowed the determination of the enantiomeric purity for all CDXs, affording values higher than 99% [149].

Considering the macrocyclic antibiotic-based CSPs, four commercially available columns were used, namely Chirobiotic T^TM^, Chirobiotic R^TM^, Chirobiotic V^TM^ and Chirobiotic TAG^TM^, under multimodal elution conditions. The optimized chiral LC conditions were successfully employed for the accurate determination of the enantiomeric purity, always higher than 99%. The studies also explored the influence of different mobile phase compositions and pH on the chromatographic parameters as well as of the structural features of the CDXs on their chiral discrimination by the macrocyclic antibiotic-based CSPs [155,156]. 

Regarding the Pirkle-type CSPs, the (*S*,*S*)-Whelk-O1^®^ CSP showed the best performance for the resolution of the CDXs evaluated, presenting very high enantioselectivity for CDXs with aromatic group linked to the chiral moiety. Polar organic elution mode presented the best chromatographic parameters allowing good resolutions and lower run time [157,158].

The overall results proved that, for the same enantiomeric pair of CDXs, the polysaccharide-based CSPs were the most efficient to separate the enantiomers of this group of compounds, since all the CDXs were enantioseparated with excellent enantioselectivity and resolution [159].

For each enantiomeric pair of the synthesized CDXs **165**–**179** the *in vitro* growth inhibitory activity in three human tumor cell lines, A375-C5 (melanoma), MCF-7 (breast adenocarcinoma), and NCI-H460 (non-small cell lung cancer), were evaluated [149]. The results obtained demonstrate that some CDXs exhibited interesting growth inhibitory effects on the tumor cell lines. The most active CDX in all human tumor cell lines was compound (**1*R***,**2*S***)-**179**. Furthermore, it is important to highlight that the effects on the growth of the human tumor cell lines were attributed not only to the nature and positions of substituents on the xanthonic scaffold, but also, in some cases, were associated with the stereochemistry of the CDXs concerning enantioselectivity results. Interesting examples of enantioselectivity were observed between the enantiomeric pairs of CDXs **165**, **167**, and **171** [149]. 

Recently, other enantioselectivity studies associated with biological activity were conducted, specifically the *in vitro* and *in silico* inhibition of cyclooxygenases (COX-1 and COX-2) for the enantiomeric pairs of CDXs **166**, **168** and **169** [160]. All CDXs exhibited COX-1 and COX-2 inhibition being, in general, the inhibitory effects similar for both COXs. The only exception was the enantiomeric pair of compound **166**, being the (*R*)-(+)-enantiomer more active at inhibiting COX-2 than COX-1. Interestingly, all pairs demonstrated enantioselectivity for COX-1. Concerning COX-2, the % of inhibition was also dependent of the stereochemistry being (*S*)-(-)-**166** and (*S*)-(+)-**169** more active [160].

Additionally, for the same enantiomeric pairs of CDXs **166**, **168** and **169**, human serum albumin (HSA) binding affinity was evaluated by spectrofluorimetry and *in silico* studies, by a docking approach [160]. All CDXs demonstrated to bind with high affinity to HSA and enantioselectivity was observed for compound **168**.

Taking into account that these CDXs have molecular moieties structurally very similar to local anaesthetics, the ability to block compound action potentials (CAP) at the isolated rat sciatic nerve was also investigated [161]. The CDXs (*S*)-**165,** (*S*)-**166** and (*S*)-**167** were chosen for biological evaluation and the results suggested that the nerve conduction blockade might result predominantly from an action on Na^+^ ionic currents. It was also investigated if the CDXs could prevent hypotonic haemolysis on rat erythrocytes. However, data suggested that all tested CDXs caused no significant protection against hypotonic when applied in concentrations high enough to block the sciatic nerve conduction in the rat [161]. 

Besides the potential as new drugs, CDXs present structural features with interest as chiral selectors for LC [23]. In this context, some of these small molecules ((*S*)-**167**, (*R*)-**168**, (*S*)-**168**, (*R*)-**176**, (1*R*,2*S*)-**179** and (1*S*,2*R*)-**179**) were selected and bound to a chromatographic support for a new application as CSPs in LC [24]. The new xanthonic CSPs afforded promising enantioresolution results, high stability and reproducibility. Accordingly, CDXs have important applications in the field of Medicinal Chemistry, not only as candidates for potential new drugs but also as analytical tools for enantioseparation in LC [37].

Recently, our group also performed enantioselectivity studies with chiral thioxanthones, *S*-analogues of xanthones, as modulators of P-glycoprotein (P-gp) [162]. It was found that one of the enantiomers modulated P-gp expression differently from its pair.

**Figure 18:**
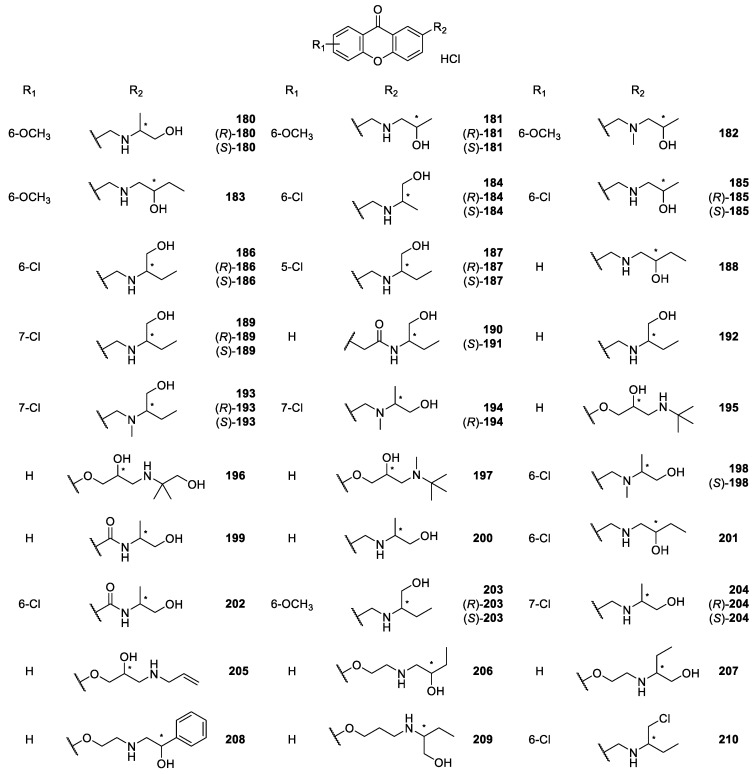
Structures of 2-aminoalkanolic CDXs **180**–**210**.

**Figure 19:**
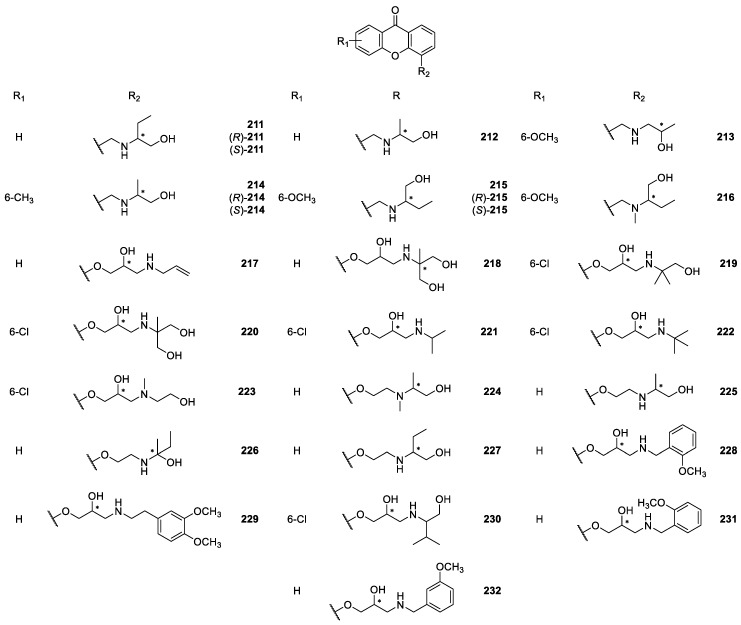
Structures of 4-aminoalkanolic CDXs **211**–**232**.

**Figure 20:**
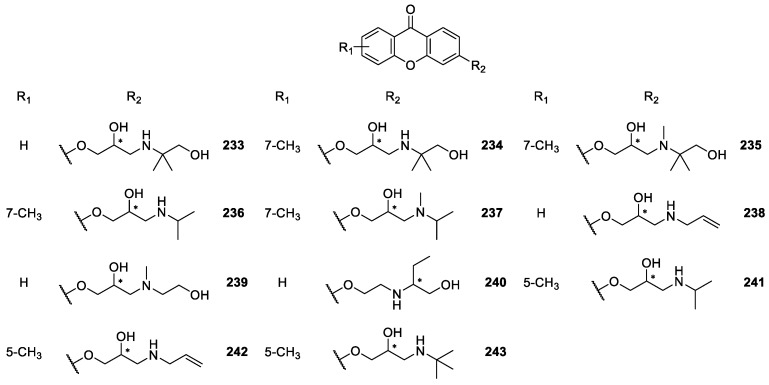
Structures of 3-aminoalkanolic CDXs **233**–**243**.

Marona *et col.* [163,164,165,166,167,168,169] also described the synthesis and biological activity evaluation of a series of aminoalkanolic CDXs **180**–**205**, **217**–**220**, **238** (Figure 18, Figure 19 and Figure 20). More recently, they synthesized the aminoalkanolic CDXs **206**–**243** (Figure 18, Figure 19 and Figure 20), being tested for anticonvulsant, antimicrobial and cardiovascular activities [170,171,172,173,174]. CDXs **180**–**194**, **198**–**202** were synthesized by condensation of an appropriate 2-bromomethylxanthone or 2-bromomethyl-7-chloroxanthone with the adequate aminoalkanol in toluene, in the presence of anhydrous potassium carbonate [163,164,175]. The exchange of secondary amino group of compound **189** for a tertiary amino group (compound **193**) was generated by reductive *N*-methylation [163]. Compound **210**, however, was synthesized through the chlorination of compound **186** with thionyl chloride in toluene [170].

Compounds **195**–**197**, **221**–**223**, **228**–**232** and **241**–**243** were synthesized by the aminolysis of 3- or 4-((oxiran-2-yl)methoxy)xanthone in *n*-propanol, or by the amination of 2-methyl-6-hydroxy-xanthone using propylene epichlorohydrin, in the presence of sodium hydroxide and water [167,172,173,174].

The synthesis of compounds **203**–**204**, **206**–**209**, **213**–**216**, **224**–**227** and **240** involved a multi-step process. At first, a substituted benzoic acid reacted with 2- or 4-methylphenol in two steps involving an Ullmann condensation and electrophilic addition. The intermediate methyl derivatives of substituted xanthone were used in the reaction with *N*-bromosuccinimide giving appropriate bromide derivatives. The last step comprised an aminolysis by means of appropriate aminoalkanol carried out in toluene in the presence of anhydrous K_2_CO_3_ [171,176].

Most of the synthesized CDXs **180**–**209**, **211**–**220**, **224**–**227**, **233**–**238**, **240** were evaluated for anticonvulsant activity [163,164,165,166,167,169,171,175,177,178,179]. The studies involved three kind of tests: maximal electroshock-induced seizures (MES), subcutaneous pentetrazole seizure threshold (scMet), and neurological toxicity (TOX).

In one of the first MES assays in mice, 2-amino-1-propanol-, 1-amino-2-propanol and 1-amino-2-butanol derivatives of 6-methoxy- or 6-chloroxanthone were the most interesting compounds. In fact, the results indicated that compound **184** was the most active [163]. Further study compared the anticonvulsant activity of CDX **184** (racemate) with the single enantiomers ((*R*)-**184**, (*S*)-**184**). All the compounds showed excellent results, and no significant differences were observed in the anticonvulsant activity of the single enantiomers compared with the racemate [166].

Additionally, the enantiomeric purity of (*R*)-**184** and (*S*)-**184** was determined by a liquid chromatography–mass spectrometry method with an electrospray ionization interface (ESI-LC/MS). The separation of the two enantiomers ((*R*)-**184** and (*S*)-**184**) was carried out on the commercially available cellulose *tris*-(3,5-dimethylphenyl carbamate) CSP, Chiralcel^®^ OD-RH, giving enantiomeric purity values higher than 99.9% [166].

Interesting anticonvulsant results were also observed with alkanolic chiral derivatives **189** and **193** of 7-chloroxanthone, which displayed anti-MES activity corresponding with that for phenytoin, carbamazepine and valproate [164]. Moreover, it is important to highlight that in this study some cases of enantioselectivity were observed. For example, although enantiomers (*R*)-**189** and (*S*)-**189** showed anticonvulsant activity, the (*S*)-enantiomer was more neurotoxic. Furthermore, considering the compound **193** (racemate), the (*R*)-enantiomer ((*R*)-**193**) in comparison to (*S*)-enantiomer ((S)-**193**) showed higher anticonvulsant activity [164,177]. Recently, several aminoalkanolic chiral derivatives substituted in positions 2, 3, or 4 evaluated for their anticonvulsant activities with 2-xanthonoxy derivatives **206, 207** and **209**, being the most active and exhibiting neurotoxicity after 30 min after administration at the dose of 100 mg/kg [171]. A further study including chiral aminoalkanol derivatives substituted in position 2 of the xanthonic scaffold (structures not shown) also emphasized the importance to examine biologically enantiomers other than racemates [178].

Additionally, a structure-anticonvulsant activity relationship study was described including series of aminoalkanol derivatives **204, 213**–**216** of 6-methoxy- or 7-chloro-2-methylxanthone as well as 6-methoxy-4-methylxanthone [176]. All the compounds showed activity in the MES screening which is recognized as one of the two most widely used seizures models for early identification of candidates as anticonvulsants. The tested compounds were evaluated in the form of racemic mixture and some additionally in the form of single enantiomers to determine stereochemistry-activity relationship. In fact, as demonstrated before [176], stereochemistry is one of the factors that can potentially influenced anticonvulsant activity of the CDXs. However, considering anti-MES activity it was not possible to establish relationship between stereochemistry and anticonvulsant properties because all sets of compounds gave different results. Racemate and enantiomers showed either similar results or diverged in duration of activity or lower effective doses. However, the anticonvulsant activity was associated with both aminoalkanol type and respective configuration as well as the location of substitution in the xanthone scaffold [176]. The overall results from several studies of Marona *et col.* [163,164,165,166,167,171,175,176,177,178,179] are quite encouraging and suggested that in the group of xanthone derivatives new potential anticonvulsants might be found.

Some of alkanolic CDXs were also evaluated for cardiovascular activity [167,173,174,175,179,180], including antiarrhythmic, hypotensive, α_1_- and β_1_-adrenergic blocking activities, effect on the normal electrocardiogram and influence on the central nervous system (CNS) [169]. Among the investigated compounds, some of them exhibited significant antiarrhythmic and/or hypotensive activity. For example, compounds **218** and **219** revealed the strongest anti-arrhythmic activity in the adrenaline-induced model of arrhythmia. Additionally, compound **219** was also the most potent concerning hypotensive activity [169]. Recently, compounds **231** and **232** were also evaluated for their cardiovascular activity, through both α_1_- and β_1_-adrenergic blocking. These compounds, classified as beta-blockers with vasodilating properties, exhibited also hypotensive vasorelaxant activities comparable to those of carvedilol [173].

The effects on platelet aggregation of racemic CDXs **180**–**182**, **195** and **196**, and the enantiomeric pure CDX (*R*)-**193** were evaluated and showed motivating results. The most active and promising compound was (*R*)-**193** which nearly completely inhibited the thrombin aggregation concentration (TAC) [181]. The results indicated that the presence of the 2-*N*-methylamino-1-butanol at position 2 and the chloride atom at the 7-position of the xanthone scaffold promoted antiplatelets activity.

Alkanolic CDXs **180**, **183**, **184** and **194** were used to assess mutagenic and antimutagenic activities in assays using the *Vibrio harveyi* test [182]. According to the obtained results, the most beneficial mutagenic and antimutagenic profiles were observed for compound **194**. This compound was shown to have strong antimutagenic activity towards the BB7 *V. harveyi* strain while failing to induce mutagenic responses in the tested strains. The modification of the chemical structure of compound **194** through chlorination of the hydroxyl group, improved considerably the antimutagenic activity maintaining the inability to induce mutagenic responses in the strains. Thus, antimutagenic potency reached a maximum with the presence of tertiary amine and one chloride atom in the side chain. Minimal activity was showed to compound **184** and no antimutagenic activity was observed for compound **180** [182].

In recent years, several aminoalkanolic CDXs **210, 228**–**230, 241**–**242** have been evaluated for their antibacterial and antifungal activities [170,172]. Compound **210** was evaluated against several dermatophytes, moulds and yeasts, exhibiting good activity results against ten strains of the *Aspergillus fumigatus, niger* and *flavus* moulds, being among the tested compounds the most active [170]. This CDX also exhibited moderate to good activity against some strains of the *Trichophyton mentagrophytes* and *rubum* dermatophytes, while being inactive against the *Candida albicans* yeast [170].

The antimicrobial activity of CDXs **228**–**230, 241**–**242** against 12 strains of the bacteria *Helicobacter pylori* was evaluated through the Kirby-Bauer method, by measuring the diameters of inhibition zones, showing that compounds **228**, **230**, **241**–**242** exhibited strong activity against the strains ATCC 43504, 700684 and 43504. Actually, those CDXs were considered the best of the tested compounds, while compound **229**, demonstrated weak antibacterial activity [172].

In a recent study on the influence of reactive oxygen species (ROS) in the anticancer activity of aminoalkanolic derivatives, it was reported that in the case of CDXs **219** and **223**, ROS were of great importance to their proapoptotic activity [183]. These encouraging results suggested that aminoalkanolic CDXs might be interesting structures for potential use in anticancer therapy [183].

#### 2.2.3. CDXs Conjuged with Amines, Amino Esters and Amino Acids

**Figure 21:**
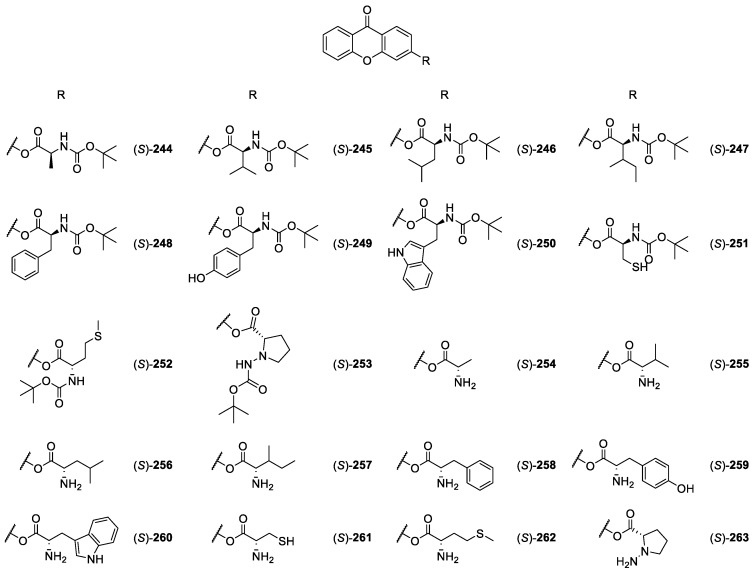
Structures of CDXs **244**–**263**, with antimicrobial and anti-inflammatory activities.

Inspired by natural xanthones properties, Rakesh et al. [184] synthesize xanthone derivatives with conjugated L-amino acids (**244**–**263**, Figure 21), to determine the corresponding antimicrobial and anti-inflammatory activities. The same research group recently reported the evaluation of *in vitro* anticancer activity of those compounds, against three different cancer cell lines, MCF-7, MDA-MB-435 and A549, validated by DNA binding and molecular docking approaches [185].

The synthetic strategy used to obtain the compounds was accomplished using 2-chlorobenzoic acid and resorcinol in anhydrous zinc chloride to give 2-chlorophenyl-(2,4-dihydroxyphenyl) methanone and cyclized with DMSO and NaOH to give 3-hydroxyxanthone. This chemical substrate was, afterwards, conjugated with different protected amino acids [184,185].

The compounds with the best antimicrobial and anti-inflammatory activities were those conjugated with L-phenylalanine, L-tyrosine and L-tryptophan, followed by compounds conjugated with L-cysteine, L-methionine and L-proline [184]. Additionally, the compounds with amino acids with high aromaticity and hydrophobicity, presented more stable amphiphilic structures. 

The antimicrobial effect comes from the penetration of the amino acid hydrophobic chains in the bacterial membranes where the cationic moiety of the amino acids interacts with the membrane phospholipids disturbing bacterial membrane. This strategy proved to be effective to develop new antimicrobial agents [184], as the microorganism die without developing mutations or resulting in loss of recognition by the antibiotics [96]. Other studies accomplished the same conclusions [168,172,186]. Regarding the antitumor activity, the compounds (*S*)-**248**, (*S*)-**249** and (*S*)-**250** exhibited potent inhibition against the tested tumor cell lines as well as DNA binding. The SAR studies showed that the aromatic and hydrophobic amino acids, such as phenylalanine, tyrosine, and tryptophan, favored the DNA binding studies and antitumor activities; whereas, aliphatic amino acids showed lower activity. The derivatives with glycine, alanine, valine, leucine, and isoleucine showed less or moderate anticancer properties [185]. 

#### 2.2.4. CDXs Containing Piperazine Moieties and Analogues

**Figure 22:**
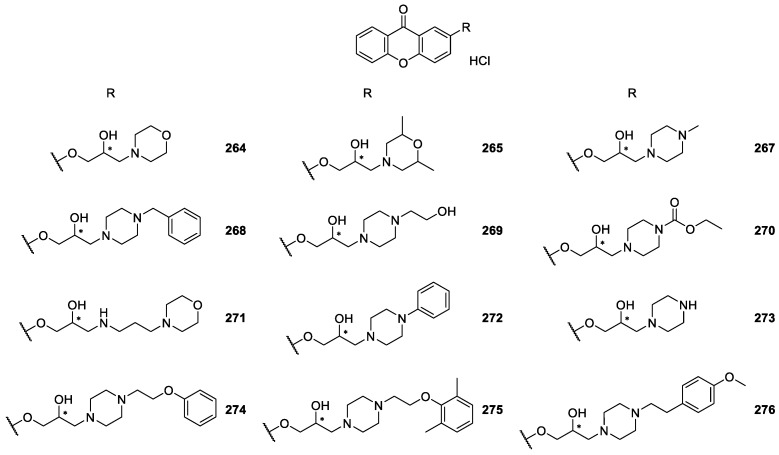
Structures of 2-piperazine derivatives **264**–**276**.

**Figure 23:**
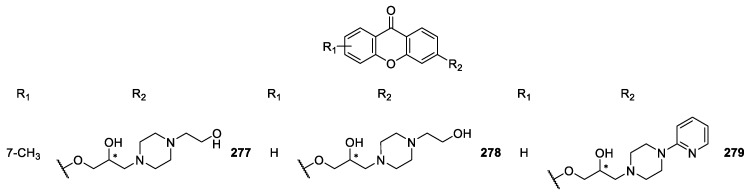
Structures of 3-piperazine derivatives **277**–**279**.

**Figure 24:**
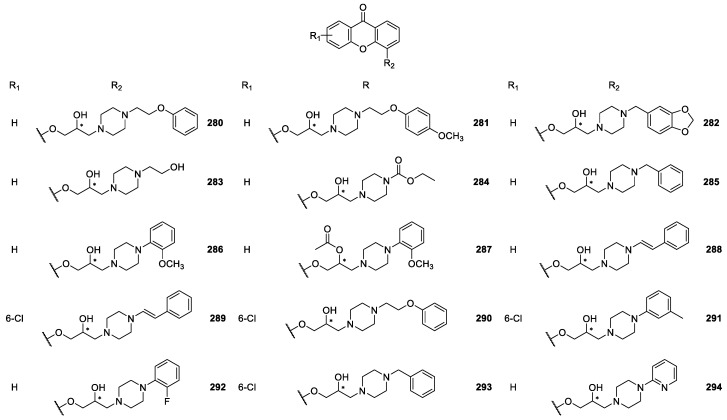
Structures of 4-piperazine derivatives **280**–**294**.

Several CDXs containing piperazine moieties (**267**–**270**, **272**–**294**) and analogues (**264**, **265**, **271**) were synthesized (Figure 22, Figure 23 and Figure 24) and their biological activity evaluated by Marona’s group [165,167,168,172,173,181,187,188,189,190,191,192].

2-Hydroxyxanthone was the building block used to synthesize compounds **267**–**273** and **277** using epichorhydrin in the presence of pyridine [165]. Compounds **274**–**276, 278, 280**–**282** were synthesized by amination of 2-(2-bromoethoxy)-9*H*-xanthen-9-one and derivatives in *n*-propanol or toluene in the presence of K_2_CO_3_ [187]. The compounds **283**–**284**, **292**–**293** were obtained by aminolysis of 4-[(2,3-epoxy)propoxy]xanthone with appropriate 1-piperazine derivatives in *n*-propanol, while **279** was synthesized using the same methodology through the aminolysis of 3-[(2,3-epoxy)propoxy]xanthone [172,173,188].

Chiral compounds **287**–**291** were obtained by amination of respective parent compounds [189] with appropriate amines in *n*-propanol. In addition, compound **287** was obtained from compound **286** by acetylation. In order to optimize synthetic methodologies, CDX **286** was obtained using an alternative method including (*R*,*S*)-4-(3-chloro-2-hydroxypropoxy)-9*H*-xanthen-9-one as intermediate [189].

CDXs **267**–**273** and **277** were evaluated for anticonvulsant activity in the MES- and subcutaneous pentylenetrazole-induzed seizures in mice and rats [165]. Among them, the most promising compound was CDX **268** which was active in both the anticonvulsant tests.

Moreover, the influence on the platelet aggregation of CDXs **264**, **265, 269** and **271** was evaluated by using adenosine-5′-diphosphate (ADP), sodium arachidonate (AA) or thrombin as the aggregating agents [181]. CDXs **265** and **271** were active, inducing 80-90% inhibition of thrombin-stimulated platelet aggregation.

Considering that the xanthone itself proved to possess vasorelaxing properties in thoracic aorta isolated from rats [193] and the strongest hypotensive effects were observed for compounds containing piperazine moiety [189], several compounds that combine the xanthone nucleus and piperazine rings (**274**–**276, 278**–**291**) were evaluated for anti-arrhythmic and/or antihypertensive activities. It is important to emphasize that CDXs **274** and **280** demonstrated to possess significant anti-arrhythmic activity in the adrenaline-induced model of arrhythmia [187]. The strongest hypotensive activity which persisted for 60 min was also associated to compound **89**.

Additionally, in another study related to the same biological activities, compound **96** was the most promising considering its effect on circulatory system. Moreover, this CDX diminished arterial blood pressure by about 40% during one hour [188].

A recent cardiovascular activity study of several CDXs **286**–**291** was described, including the following pharmacological experiments: the binding affinity for adrenoceptors, the influence on the normal electrocardiogram, the effect on the arterial blood pressure and prophylactic antiarrhythmic activity in adrenaline induced model of arrhythmia (rats, iv) [189]. The CDXs **286** and **287** revealed to act as potential antiarrhythmics in adrenaline induced model of arrhythmia in rats after intravenous injection. In another study, CDX **279** was reported as a promising hypotensive with its activity attributed to the blockage of α1-adrenoreceptors [173]. The results obtained were quite encouraging and suggested that in the group of xanthone derivatives new potential antiarrhythmics and hypotensives might be found.

A series of some chiral derivatives of 2-xanthones **267**–**273, 277** with a piperazine moiety was evaluated for their activity against *M. tuberculosis*. The highest level of activity against *M. tuberculosis* was observed for compound **270**, which exhibited 94% growth inhibition. This compound was also examined for its anti-*M. avium* activity as well as cytotoxicity, showing insignificant anti *M. avium* activity and cytotoxic effects [188]. 

Recently, CDXs **284**–**285, 290, 292, 293** were evaluated for their antibacterial activity against 12 strains of the bacteria *Helicobacter pylori* [172]. CDX **285** showed strong activity against *H. pylori* strain ATCC 43504 and 700684, being the only compound to show higher activity against clarithromycin-resistant *H. Pylori* strains, than to the one resistant to metronidazole [172]. 

#### 2.2.5. CDXs Containing Other Moieties

**Figure 25:**
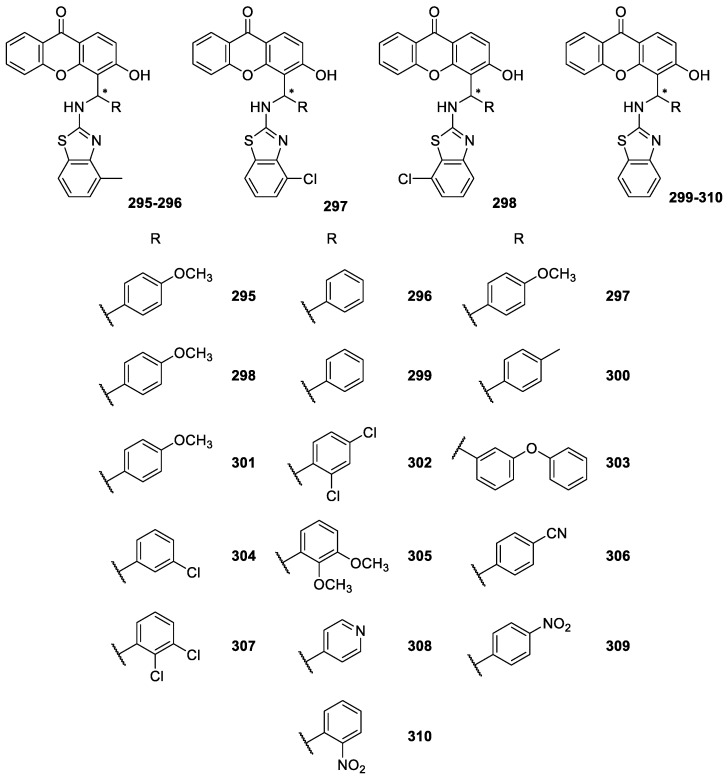
Structures of CDXs **295**–**310**.

Recently, Cherkadu et al. [194] reported the synthesis of some CDXs **295**–**310** (Figure 25) containing moieties other than piperazine and aminoalcohols [194]. The synthesis of these CDXs, a series of 2-(aminobenzothiazol)methylxanthones, was performed through the reaction of 3-hydroxy-xanthone, aromatic aldehydes and 2-aminobenzothiazoles in DMF at 120°C with FeCl_3_ as catalyst [194]. To the best of our knowledge, no biological activities were reported for those CDXs.

A summary of the synthetic CDXs obtained by binding/coupling chiral moieties to the xanthone scaffold, their biological activities as well as the associated references are presented in Table 2.

## 3. Conclusions

The synthetic CDXs, inspired in natural sources, and obtained by coupling chiral moieties to the xanthone scaffold, demonstrated potential to perform a large variety of biological activities, including antitumor, antimicrobial, anticonvulsant, antimalarial, anti-inflammatory, antiplatelet, anti- thrombotic, antipyretic, analgesic, antioxidant, antidiabetic, anticoagulant, among others. Nevertheless, for this family of compounds the main biological activities reported were antitumor and antimicrobial.

The more studied chiral moieties were amines, amino alcohols and amino acids. The cationic moieties of the amino acids have been indicated as a good approach to develop new antimicrobial agents both for CDXs inspired in natural xanthones and obtained by coupling chiral moieties to the xanthone scaffold. Regarding enantioselectivity, some studies reported the importance in SAR studies, but the majority neglected the influence of stereochemistry in the biological activity.

## Figures and Tables

**Table 1:** Summary of the biological activities of synthetic CDXs inspired in natural xanthones.

CDXs	Biological Activities	Ref.
Kielcorin derivatives **2**–**9**	Antitumor and protein kinase C inhibition	[56,58,59,61]
Psorospermin derivatives **11**–**15**	Antitumor	[64]
Muchimangin derivatives **16**–**20**	Antimicrobial	[66]
Mangiferin derivatives **21**–**57**	Antipyretic, antimicrobial, analgesic, antioxidant, anti-inflammatory, antidiabetic, anticoagulant and antiplatelet	[68,72,73,75,76,77,78,81,82,83,86]
α-Mangostin derivatives **58**–**70**	Antimicrobial, hemolytic and antimycobacterial	[91,95,96,97,98,99,118]
Caged xanthones **71**–**155**	Antimalarial, antitumor, anti-proliferation and anti-angiogenesis	[106,108,109,111,113,114,115,116,117,119,120]

**Table 2:** Summary of the biological activities of synthetic CDXs obtained by binding/coupling chiral moieties to the xanthone scaffold.

CDXs	Biological Activities	Ref.
XAA, DMXAA and analogues **156**–**164**	Antitumor,	[122,123,124,125,126,127,128,129,130,131,132,133,136]
	antiviral,	[134]
	antiplatelet, antithrombotic,	[135]
	anti-inflammatory	[137]
	and analgesic	[140]
Aminoalkanolic CDXs **165**–**243**	Cyclooxygenases inhibition,	[160]
	antitumor,	[148]
	anticonvulsant,	[139,140,141,142,143,144,145,146,147,148,149,150,151,152,153,154,155,156,157,158,159,160,161,162,163,167,171,175,176,177,178,179]
	cardiovascular,	[167,169,173,174,175,179,180]
	antiplatelet aggregation,	[181]
	antimutagenic,	[182]
	antifungal,	[170]
	antibacterial and	[172]
	anticancer	[183]
CDXs conjugated with amines, amino esters and amino acids **244**–**263**	Anti-inflammatory	[168,172,186]
CDXs containing piperazine moieties and analogues **264**–**294**	Anticonvulsant,	[165]
	antiplatelet aggregation,	[181]
	cardiovascular,	[173,187,188,189,190]
	antifungal and	[188]
	antibacterial	[172]
CDXs containing other moieties **295**–**310**	No activities reported	[194]
